# Active hexose-correlated compound enhances extrinsic-pathway-mediated apoptosis of Acute Myeloid Leukemic cells

**DOI:** 10.1371/journal.pone.0181729

**Published:** 2017-07-20

**Authors:** Kavin Fatehchand, Ramasamy Santhanam, Brenda Shen, Ericka L. Erickson, Shalini Gautam, Saranya Elavazhagan, Xiaokui Mo, Tesfaye Belay, Susheela Tridandapani, Jonathan P. Butchar

**Affiliations:** 1 Medical Scientist Training Program, The Ohio State University, Columbus, Ohio, United States of America; 2 Department of Internal Medicine, The Ohio State University, Columbus, Ohio, United States of America; 3 Center for Biostatistics, The Ohio State University, Columbus, Ohio, United States of America; 4 School of Arts and Sciences, Bluefield State University, Bluefield, WV, United States of America; European Institute of Oncology, ITALY

## Abstract

Active Hexose Correlated Compound (AHCC) has been shown to have many immunostimulatory and anti-cancer activities in mice and in humans. As a natural product, AHCC has potential to create safer adjuvant therapies in cancer patients. Acute Myeloid Leukemia (AML) is the least curable and second-most common leukemia in adults. AML is especially terminal to those over 60 years old, where median survival is only 5 to 10 months, due to inability to receive intensive chemotherapy. Hence, the purpose of this study was to investigate the effects of AHCC on AML cells both *in vitro* and *in vivo*. Results showed that AHCC induced Caspase-3-dependent apoptosis in AML cell lines as well as in primary AML leukopheresis samples. Additionally, AHCC induced Caspase-8 cleavage as well as Fas and TRAIL upregulation, suggesting involvement of the extrinsic apoptotic pathway. In contrast, monocytes from healthy donors showed suppressed Caspase-3 cleavage and lower cell death. When tested in a murine engraftment model of AML, AHCC led to significantly increased survival time and decreased blast counts. These results uncover a mechanism by which AHCC leads to AML-cell specific death, and also lend support for the further investigation of AHCC as a potential adjuvant for the treatment of AML.

## Introduction

Acute Myeloid Leukemia (AML) is the most common type of acute leukemia in adults, causing over 10,500 deaths and affecting over 20,000 people in 2015 [1–3]. This disease is characterized by an infiltration of the bone marrow, blood, and other tissues by myeloid precursors, or “blasts,” which are unable to differentiate [4]. Despite multiple biologically-distinct subtypes of AML, the current methodology of treatment includes a regimen of chemotherapy and stem cell transplant [5]. AML is especially aggressive to those over 60 years old, where median survival is only 5 to 10 months due to the inability to receive intensive chemotherapy [5,6]. The M3 subtype of AML, known as Acute Promyelocytic Leukemia (APL), is treatable with *All-trans-retinoic acid (ATRA)*, as it promotes cell differentiation leading to decreased myeloid precursors within the patient [7]. However, the other subtypes of AML appear refractory to this drug and therefore represent an urgent need for newer treatment strategies.

Cancer immunotherapies and adjuvants can help stimulate the immune system to respond more effectively against tumors and tumor cells. Such immune modulators, which include recombinant cytokines such as Interferons, synthetic compounds such as Toll-like receptor agonists, and natural products containing immune-stimulatory molecules, are being explored as potential enhancers of antibody therapy [8–11]. Since 2007 there have been 12 novel natural products that have been brought to market including Ixabepilone (Ixempra ®) for aggressive breast cancer and Vinflunine (Javlor ®) for bladder cancer [12].

Active Hexose-Correlated Compound (AHCC) is a mushroom extract derived from several species of *Basidiomycetes* mushrooms including Shiitake (*Lentinus edodes*) and Shimeji (*Lyophyllum shimeji*) [13]. This natural product is composed of a mixture of amino acids, minerals, polysaccharides and lipids enriched in α-1,4-linked glucans [14–16]. AHCC is used as a nutritional supplement in Japan and has been shown to be effective against hyperlipidemia, obesity and cancer [14]. AHCC is an immunostimulatory agent [13,17,18] and has improved the prognosis and quality of life of patients with liver, lung, and head and neck cancer [19–21]. Here, we sought to examine the potential for AHCC as a treatment against AML. We found that treatment of AML cell lines and primary AML leukopheresis samples with AHCC led to an increase in apoptosis, which was Caspase-3-dependent. Additionally, treatment with AHCC induced both extrinsic apoptotic pathway members Fas and Caspase-8. In a mouse engraftment model of AML, AHCC led to reduced blast counts and increased survival time. These results uncover a mechanism of AHCC-induced AML cell death, and also suggest that further study of AHCC as a possible AML therapeutic may be warranted.

## Materials and methods

### Cell culture

The AML cell lines used in this study (MV-4-11, MOLM-13, OCI-AML3 and THP-1) was purchased from the ATCC and cultured according to ATCC recommendations. Cells were maintained below 1 x 10^6^ cells/mL in RPMI 1640 media (Gibco, Grand Island, NY) supplemented with 10% heat-inactivated fetal bovine serum (FBS; Hyclone Laboratories, Grand Island, NY), 2mM L-glutamine (Invitrogen, Grand Island, NY), and penicillin/streptomycin (56 U/mL/56 μg/mL; Invitrogen) at 37°C in an atmosphere of 5% CO_2_. HS-5 stromal cells were generously provided by Shelley Orwick and Dr. John C. Byrd (The Ohio State University, Columbus, OH) and were cultured as described above.

### Primary cells

Primary cell handling was done as described previously.[22] White blood cells apheresed from AML patients were obtained after written informed consent in accordance with the Declaration of Helsinki under a protocol approved by the institutional review board of The Ohio State University. Cells were stored in liquid nitrogen in 20% FBS and 10% DMSO until needed for experiments. At the time of the experiment, cells were thawed at 37°C and incubated in RPMI 1640 media (Gibco) supplemented with 20% FBS, 2mM L-glutamine (Invitrogen) and penicillin/streptomycin (56 U/mL/56 μg/mL; Invitrogen) at 37°C in an atmosphere of 5% CO_2_ for 1 hour. Cells were then centrifuged and maintained at 3 x 10^6^ cells/mL in RPMI 1640 media (Gibco) supplemented with 20% FBS, 2mM L-glutamine (Invitrogen) and penicillin/streptomycin (56 U/mL/56 μg/mL; Invitrogen) and were either left untreated or treated with increasing doses of AHCC (0, 1, 5, 10 mg/mL) (Quality of Life Labs LLC, Purchase, NY) and incubated for 24 hours at 37°C. The next day, cells were counted using Trypan blue exclusion and used for assays.

### Antibodies

Anti-Caspase-3, Anti-Caspase-8, Anti-Caspase-9, and anti-PARP antibodies for Western blotting were purchased from Cell Signaling Technology (Danvers, MA). Anti-Calreticulin antibody was purchased from Enzo Life Sciences (Farmingdale, NY). Anti-rabbit and anti-mouse HRP conjugated secondary antibodies were purchased from Cell Signaling Technology (Danvers, MA). For cell viability assays, Annexin V FITC/propidium iodide (BD Biosciences) was used following the protocol of the manufacturer.

### Colony forming assay

MV4-11 cells were treated with increasing doses of AHCC (0, 1, 5, 10 mg/mL) for 24 hours then plated at 1x10^3^ in duplicate, in 0.9% methylcellulose medium (Methocult H4100, Stem Cell Technologies) on cell culture plates for 2 weeks. Colonies were then scored in a double-blind fashion.

### Western blotting

Western blotting was done as described previously.[23] Cells were lysed in TN1 buffer (50 mM Tris (pH 8.0), 10 mM EDTA, 10 mM Na_4_P_2_O_7_, 10 mM NaF, 1% Triton X-100, 125 mM NaCl, 10 mM Na_3_VO_4_, and 10 μg/ml each aprotinin and leupeptin). Protein lysates were boiled in Laemmli sample buffer, separated by SDS-PAGE, transferred to nitrocellulose membranes, probed with the antibody of interest, and then developed by Pierce ECL 2 Western blotting substrate (Thermo Scientific, Rockford, IL) or SuperSignal West Femto maximum sensitivity substrate (Thermo Scientific). Densitometry was performed using ImageJ, normalizing bands in each lane to loading control to generate the bar graphs.

### Real-time polymerase chain reaction

Total RNA was isolated using the Total RNA Purification Plus Kit (Norgen Biotek Corporation, Ontario, Canada). RNA was reverse transcribed and subjected to quantitative real-time (qRT)–PCR using Power SYBR Green Master Mix (Applied Biosystems, Grand Island, NY). The following primers (Invitrogen) were used: GAPDH (forward primer 5′-ATT CCC TGG ATT GTG AAA TAG TC-3′ and reverse primer 5′-ATT AAA GTC ACC GCC TTC TGT AG-3′); Fas/CD95 (forward primer 5’-AAG ACT GTT ACT ACA GTT G-3’ and reverse primer 5’-GCT TAT GGC AGA ATT GGC CA-3’); TRAIL (forward primer 5’-AAG GCT CTG GGC CGC AAA ATA AAC-3’ and reverse primer 5’-GCC AAC TAA AAA GGC CCC GAA AAA-3’); TRAIL-R1 (forward primer 5’-CAG AAC GTC CTG GAG CCT GTA AC-3’ and reverse primer 5’-ATG TCC ATT GCC TGA TTC TTT GTG-3’); TRAIL-R2 (forward primer 5’-GGG AAG AAG ATT CTC CTG AGA TGT G-3’ and reverse primer 5’-ACA TTG TCC TCA GCC CCA GGT CG-3’). GAPDH was used for normalization of the genes of interest. Relative copy number (RCN) was calculated as *2*^–ΔCt^ × 100 (52), where 0078Ct is the Ct_(target)_ –Ct_(GAPDH)_. RCN was then normalized to calculate fold-change versus untreated.

### AHCC preparation for experiments *in vitro*

AHCC was purchased from Quality of Life Labs LLC (Purchase, NY). Following de-waxing and lyophilization (according to manufacturer instructions), AHCC was freshly prepared by dissolving into PBS at a final concentration of 100 mg/mL. After dissolving, the solution was passed through a 0.22-micron filter (Millipore, Billerica, MA) and used immediately, at up to 10 mg/ml [24].

### AML murine model

All animal experiments were done in full accordance with a protocol approved by the Institutional Animal Care and Use Committee (IACUC) at The Ohio State University. Female non-obese diabetic severe combined immunodeficient-γ (NSG) mice were purchased from Jackson ImmunoResearch Laboratories (Ban Harbor, ME) and bred within a campus-located vivarium under the direction of Dr. Adrienne Dorrance (Division of Hematology, The Ohio State University). Splenocytes from MV4-11-engrafted mice (0.3x10^6^ resuspended in PBS) were intravenously injected into the tail vein of 6-week-old NSG mice. After one week, mice received either AHCC (600 mg/kg) mixed into PBS, or PBS control by gavage twice per week for 2 weeks. Similar doses of AHCC were used previously as daily treatments with no evidence of toxic effects [13,14,25,26]. Gavage was performed by using a plastic feeding tube (Instech Laboratories, Inc, Plymouth Meeting, PA). Survival was measured as the time before meeting early-removal criteria set within the protocol, which included 20% weight loss, paralysis or inability to stand, uncontrolled shivering, or unwillingness to eat or drink.

### Cell survival assay

AML cells were treated with increasing doses of AHCC (0, 1, 5, 10 mg/ml) for 24 or 48 hours. Cells were either subjected to Trypan Blue (Sigma St. Louis, MO) or harvested and stained with Annexin V FITC/propidium iodide (BD Biosciences) using the protocol of the manufacturer.

### Statistics

Cell-line data were analyzed by analysis of variance (ANOVA). For the experiments using healthy-donor or AML-patient samples, since the same sample was under different treatment conditions, data were analyzed by mixed-effect models. For the mouse experiment, the probabilities of disease development were compared between groups using a log-rank test, and the white-blood-cell (WBC) counts analyzed by mixed-effect modeling. Holm’s method was used to adjust for multiplicity.

## Results

### AHCC decreases survival of AML cells

**Fig 1:**
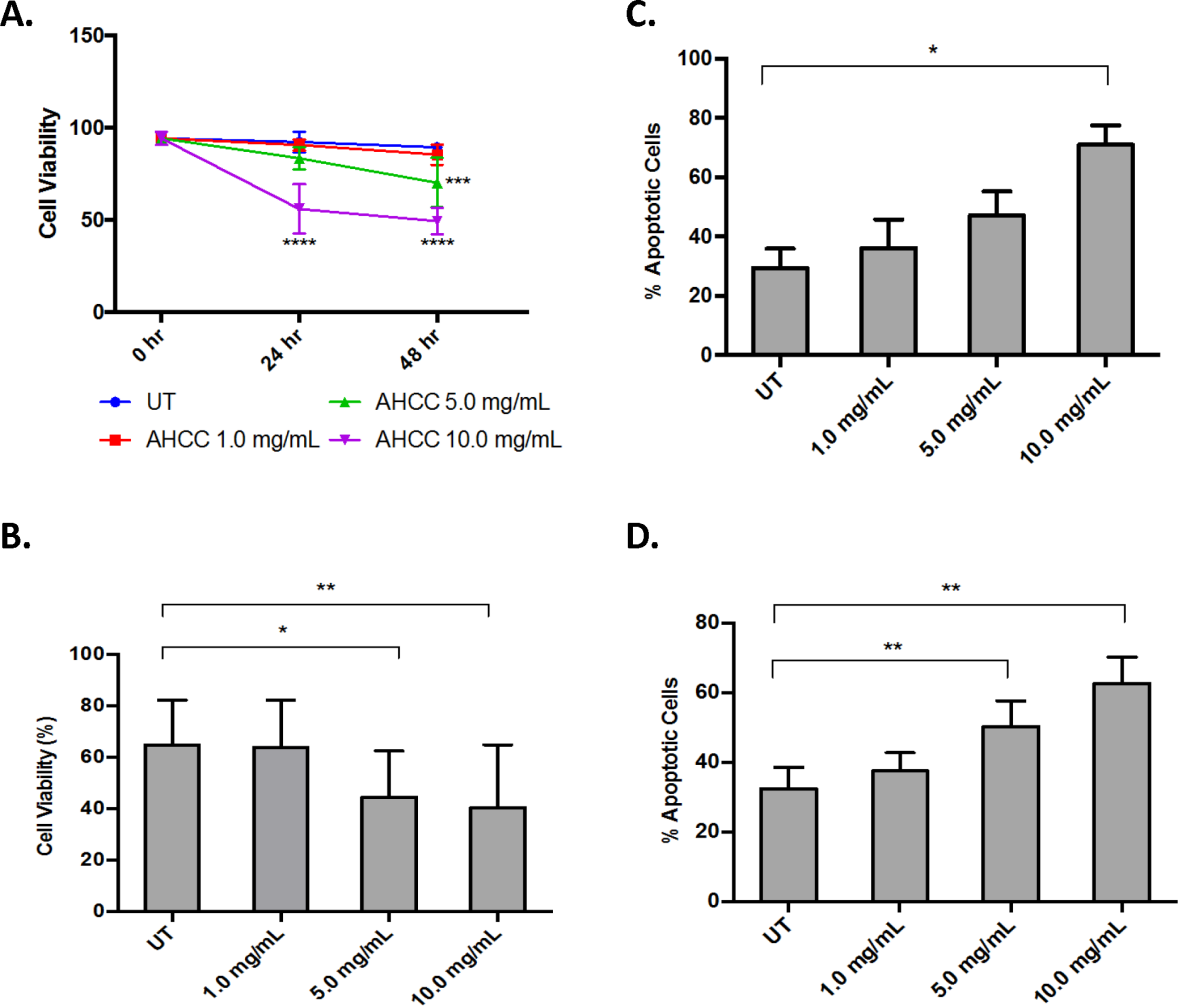
AHCC decreases survival of AML cells. The AML cell line MV4-11 (1 x 10^6^ cells/ml) and primary AML-patient leukopheresis samples (3 x 10^6^ cells/ml) were treated with 0, 1, 5 or 10 mg/ml AHCC for 24 or 48 hours. Trypan Blue Exclusion was done with (A) MV4-11 cells (n = 3 separate experiments) and (B) primary AML leukopheresis samples (n = 7 donors). (C-D). MV4-11 (C, n = 3 separate experiments) and patient leukopheresis samples (D, n = 7 donors) were treated as above and then analyzed via flow cytometry following Annexin V and Propidium Iodide (PI) staining. * p≤0.05; ** p≤0.01.

**Fig 2:**
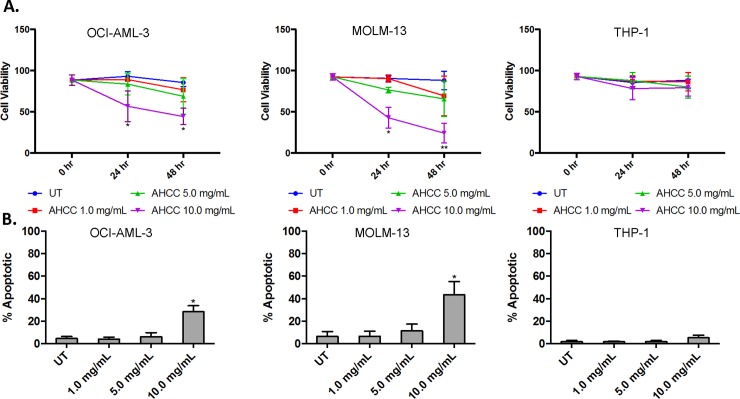
AHCC increases apoptosis in most AML cell lines. The AML cell lines OCI-AML3 (left panels, 1 x 10^6^ cells/ml), MOLM-13 (middle panels, 1 x 10^6^ cells/ml) and THP-1 (right panels, 1 x 10^6^ cells/ml) were treated with 0, 1, 5 or 10 mg/ml of AHCC for 24 or 48 hours. (A). Trypan Blue Exclusion was done to measure viability (n = 3 separate experiments). (B). The 3 respective cell lines were treated as above and Annexin V / Propidium Iodide (PI) staining was measured using flow cytometry (n = 3 separate experiments). * p≤0.05.

Because AHCC can activate monocytes and monocytic cell lines [13,26,27] and because AML blasts are immature myeloid-lineage cells, we sought to determine whether AHCC could directly affect blast-cell survival and proliferation. We began by testing AHCC against the MV4-11 cell line, which contains the FLT3-ITD mutation shared by approximately 20% of AML patients [28] and is linked to increased risk of relapse and mortality [29]. We treated MV4-11 cells with AHCC (concentrations from 0 to 10 mg/ml) and measured cell viability. Results showed that 10 mg/ml of AHCC significantly reduced viability at 24 and 48 hours (Fig 1A). To determine whether this involved apoptosis, we treated MV4-11 cells with increasing concentrations of AHCC. Annexin V and Propidium Iodide (PI) staining showed significantly higher apoptosis in treated MV4-11 cells (Fig 1C). We repeated this using primary patient samples and found that 5 mg/ml of AHCC was sufficient to significantly increase apoptosis (Fig 1D). To supplement this we also tested the AML cell lines OCI-AML3, MOLM-13 and THP-1. Results showed that AHCC decreased the viability of OCI-AML3 and MOLM-13 cells, but not THP-1 (Fig 2A). Similarly, Annexin/PI staining showed that AHCC led to apoptosis in OCI-AML3 and MOLM-13 but not in THP-1 cells (Fig 2B).

These results show that 3 of 4 cell lines and all tested patient samples are sensitive to AHCC treatment. However, AML blasts typically rely on stromal-cell support [30], and the lack of this support in our culture conditions may have played a role. To address this we tested the effects of AHCC on co-cultures of primary AML samples and HS-5 stromal cells, finding that the stromal cells had no effect on AHCC-induced reductions in viability and increased apoptosis (data not shown).

### AHCC decreases AML-cell proliferation

**Fig 3:**
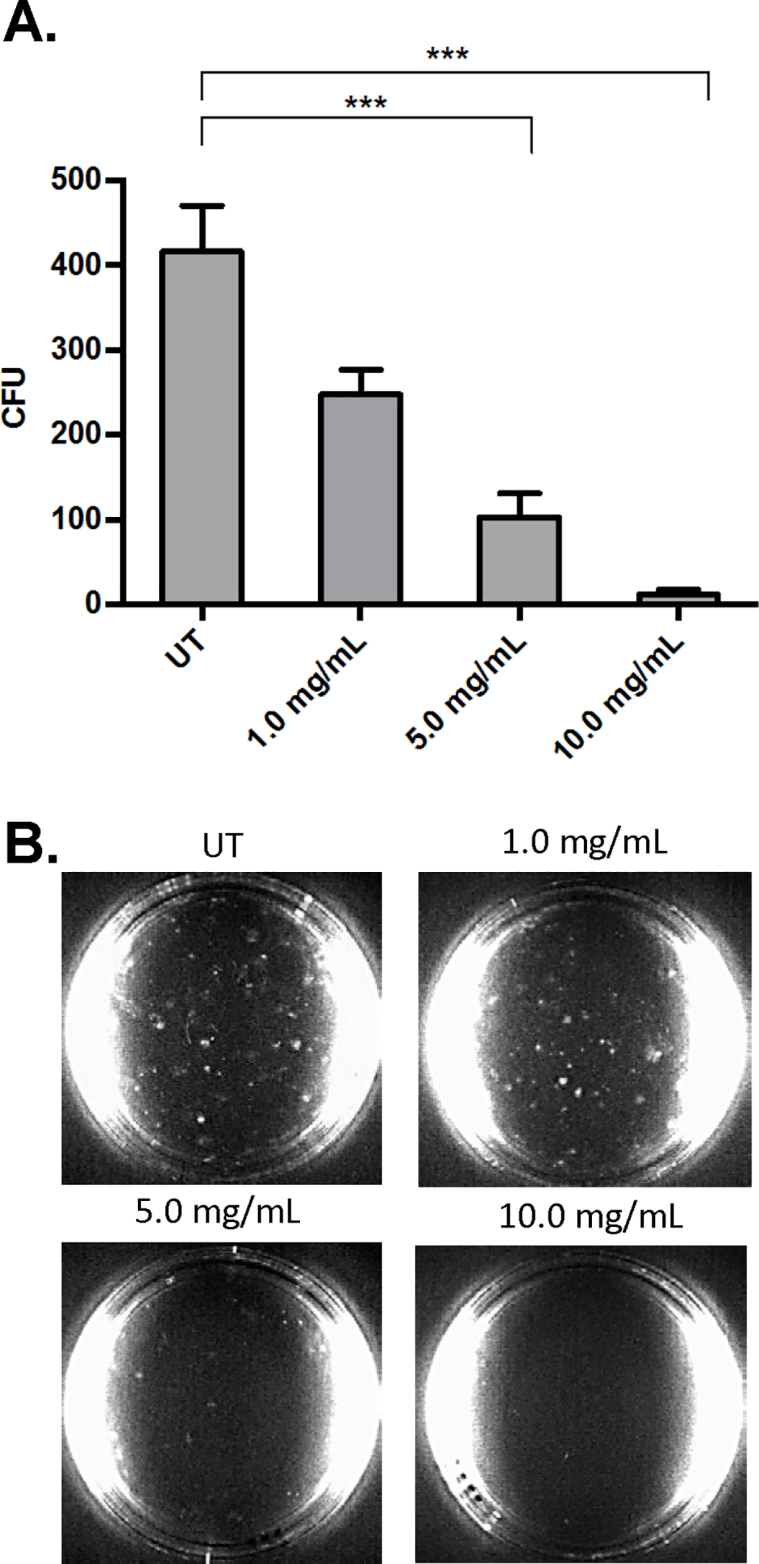
AHCC decreases AML-cell proliferation. MV4-11 cells (1 x 10^6^ cells/ml) were treated with AHCC as in Fig 1, then incubated on Methocult-media-containing plates for 2 weeks. Colonies were counted in a blinded fashion. (A). Graph of 3 separate experiments. (B). Photographs of representative plates. *** p≤0.001.

To test the effects of AHCC on blast-cell proliferative ability, we treated MV4-11 cells with increasing concentrations of AHCC and plated them on methocult media-containing plates for 2 weeks. Following this, colony formation was counted in a double-blinded fashion and results showed significantly fewer colonies with increasing doses of AHCC (Fig 3A and 3B).

### AHCC-induced cell death is Caspase-3-dependent

**Fig 4:**
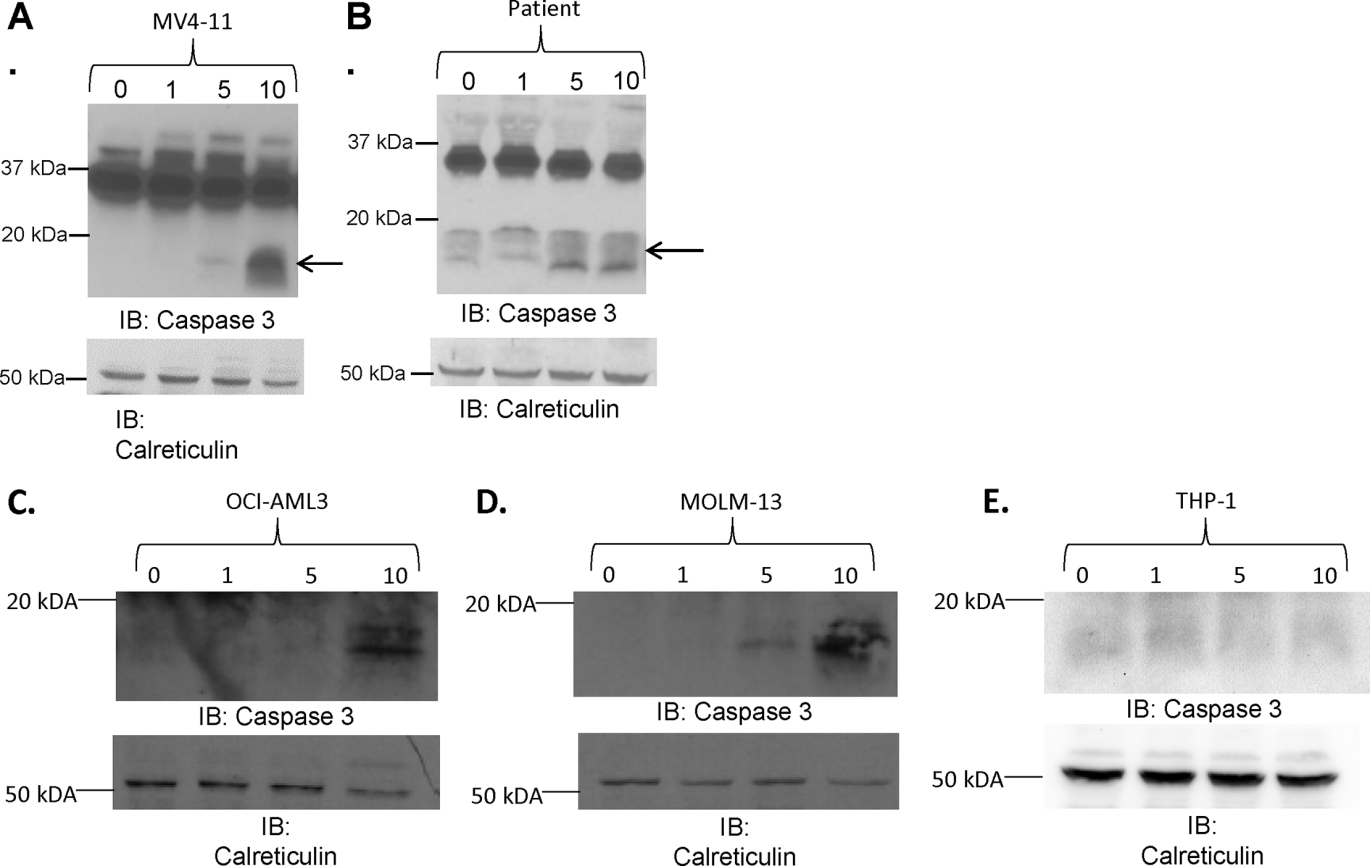
AHCC-induced cell death is Caspase-3-dependent. The AML cell line MV4-11 (1 x 10^6^ cells/ml) and primary AML-patient leukopheresis samples (3 x 10^6^ cells/mL) were treated with 0, 1, 5 or 10 mg/ml AHCC for 24 hours. (A-B). Western blotting was done to measure cleaved Caspase-3, using Calreticulin as a loading control. Representative blots are shown for MV4-11 (A, n = 3 separate experiments) and primary leukopheresis samples (B, n = 3 donors). (C-E). OCI-AML3 (C), MOLM-13 (D) and THP-1 (E) cells were treated with AHCC as above. Western blotting was done to measure cleaved Caspase-3, using Calreticulin as a loading control (n = 3 separate experiments).

Caspases are vital mediators of both the extrinsic and intrinsic apoptotic pathways. Caspase-3 plays an especially crucial role as a death protease activated either by tumor necrosis factor (TNF) family receptors, FADD, and Caspase-8 in the extrinsic pathway, or via the intrinsic pathway involving mitochondrial release of cytochrome c and Apaf-1-mediated processing of Caspase-9. Following such extrinsic or intrinsic activation, Caspase-3 can then act to cleave a battery of substrates and thereby initiate apoptotic processes [31]. To test whether Caspase-3 played a role in AHCC-induced AML-cell death, we treated all four AML cell lines and primary AML leukopheresis cells with increasing concentrations of AHCC (0, 1, 5 or 10 mg/ml) for 24 hours and measured cleaved Caspase-3. Results showed that higher doses of AHCC induced Caspase-3 cleavage in MV4-11 (Fig 4A) and primary patient cells (Fig 4B). In concordance with results seen with apoptosis, Caspase-3 cleavage was seen with OCI-AML3 and MOLM-13 cells (Fig 4C and 4D) but not THP-1 cells (Fig 4E).

**Fig 5:**
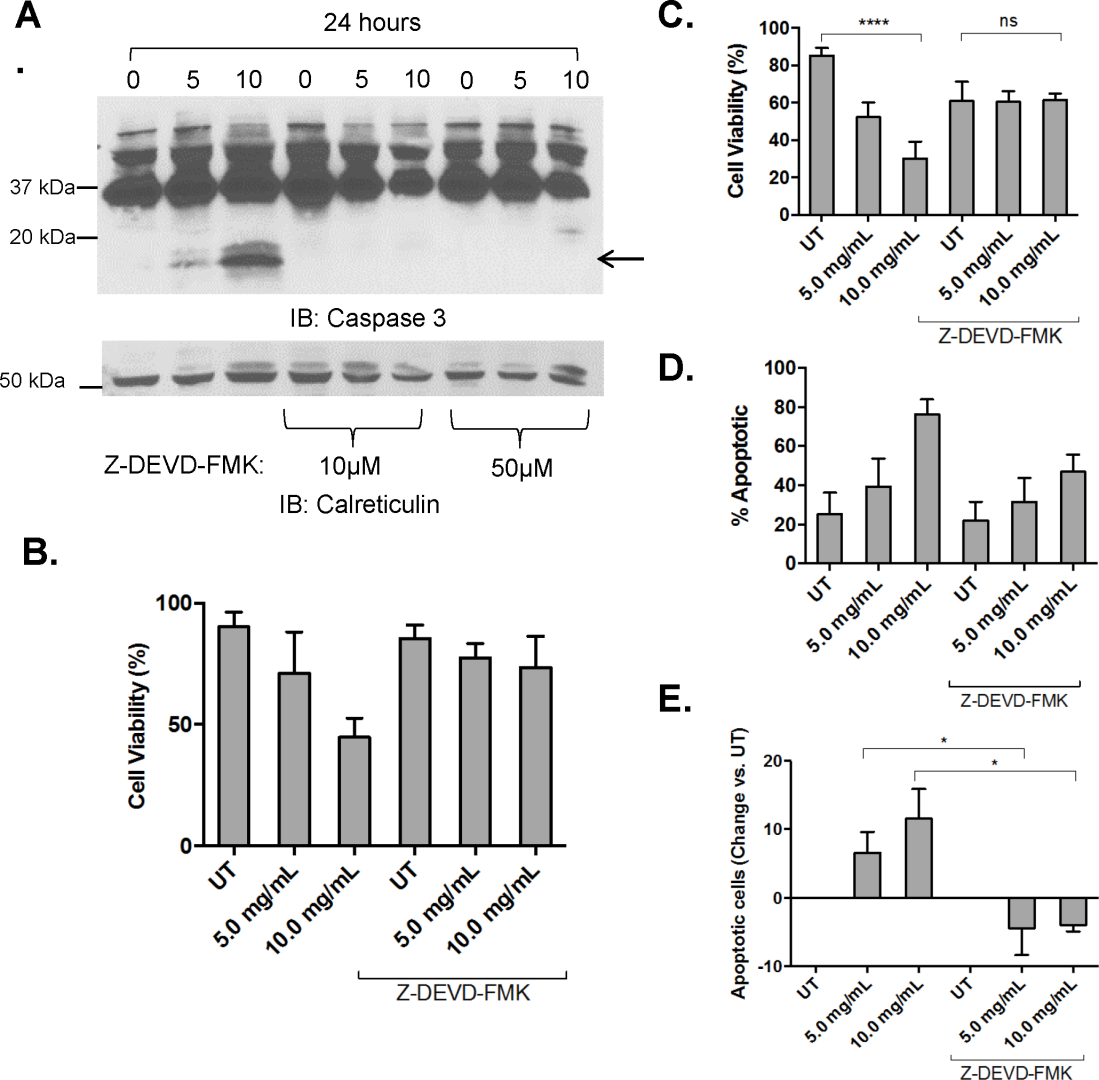
Caspase-3 cleavage is required for AHCC-induced apoptosis of AML cells. (A). MV4-11 cells (1 x 10^6^ cells/ml) were treated with 0, 1, 5 or 10 mg/ml AHCC for 24 hours in the presence or absence of 50 μM Z-DEVD-FMK, the Caspase-3 inhibitor. Western blotting was done to measure cleaved Caspase-3, with Calreticulin as loading control (n = 2 separate experiments, representative blots shown). (B-C). MV4-11 cells (B, n = 2 separate experiments) and primary leukopheresis samples (C, n = 3 donors) were treated for 24 hours with 0 (UT), 5 or 10 mg/ml AHCC with or without Caspase-3 inhibitor, followed by Trypan Blue counts to measure viability. (D-E). MV4-11 cells (D, n = 2 separate experiments) and primary leukopheresis samples (E, n = 3 donors) were treated for 24 hours with 0 (UT), 5 or 10 mg/ml AHCC with or without Caspase-3 inhibitor and Annexin V / Propidium Iodide (PI) staining measured by flow cytometry. * p≤0.05; **** p≤0.0001.

Next, to test the involvement of cleaved Caspase-3 in AML-cell death, we pretreated MV4-11 cells and primary AML leukopheresis samples with a Caspase-3 inhibitor, Z-DEVD-FMK for 45 minutes, and treated with increasing concentrations of AHCC for 24 hours. Inhibitor efficacy was confirmed by measuring Caspase-3 cleavage (Fig 5A). Cell viability was measured, and results from Trypan Blue (Fig 5B & 5C for MV4-11 and primary AML cells, respectively) and Annexin V and Propidium Iodide (PI) staining (Fig 5D & 5E for MV4-11 and primary AML cells, respectively) showed that the Caspase-3 inhibitor ameliorated the apoptotic effect of AHCC. This suggests that the pro-apoptotic effects of AHCC on AML cells are mediated by Caspase-3.

## AHCC induces Caspase-8 cleavage and upregulation of Fas and TRAIL

Since AHCC induced Caspase-3 cleavage in three out of four AML cell lines, we next asked which upstream molecules were involved in the induction of Caspase-3 cleavage. Both the extrinsic and intrinsic apoptotic pathways may be involved in Caspase-3 cleavage, so we chose to look at the intrinsic apoptotic molecule Caspase-9 and the extrinsic molecule Caspase-8. Caspase-9 is an initiator caspase involved in intrinsic apoptosis. Upon apoptotic stimulation, cytochrome c is released from the mitochondria, which forms a complex with pro-Caspase-9 and Apaf-1. This results in the cleavage and activation of Caspase-9 which can then activate other caspases including Caspase-3 [32]. In the extrinsic apoptotic pathway, death receptors can activate Caspase-8 through their interaction with adaptor proteins. Active Caspase-8 or the p18 subunit is the first step in the apoptotic signaling cascade, which eventually leads to Caspase-3 cleavage, and apoptosis [33–35].

**Fig 6:**
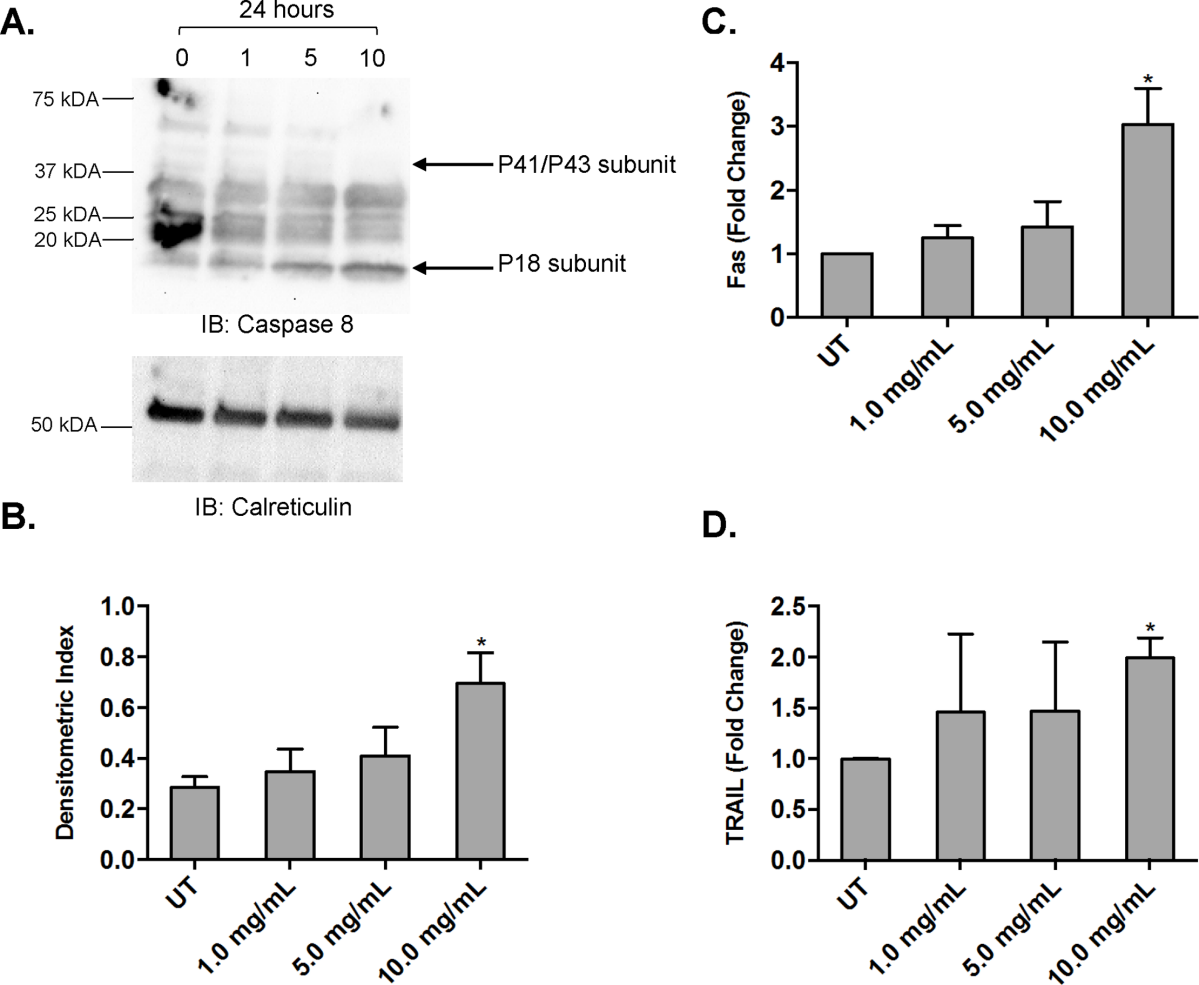
AHCC induces Caspase-8 cleavage and upregulation of Fas and TRAIL. The AML cell line MV4-11 (1 x 10^6^ cells/ml) was treated with 0, 1, 5 or 10 mg/ml AHCC for 24 hours. (A-B). Western blotting was done to measure cleaved Caspase-8, with Calreticulin as a loading control (A, representative blot shown), and densitometric analysis was performed (B, n = 3 separate experiments). (C-D). Fas (C, n = 3 separate experiments) and TRAIL (D, n = 4 separate experiments) were measured by qPCR. * p≤0.05.

Here, we treated MV4-11 cells with increasing doses of AHCC (0, 1, 5, 10 mg/ml) for 24 hours and measured levels of cleaved Caspase-8 and Caspase-9. Results showed that AHCC induced the cleavage of Caspase-8 (Fig 6A and 6B) but not Caspase-9 (data not shown) in MV4-11 cells.

This suggested involvement of the extrinsic apoptotic pathway so we next tested whether the death receptors Fas or tumor necrosis factor (TNF)-related apoptosis inducing ligand receptor (TRAILR) increased upon AHCC stimulation. We treated MV4-11 cells as described above and measured TRAIL-R1, TRAIL-R2 and Fas by qPCR. Results showed that the death receptor Fas increased with higher concentrations of AHCC (Fig 6C), whereas TRAIL-R1 and TRAIL-R2 did not change (data not shown). Similarly we examined the effects of AHCC on PARP and saw no increase in the cleaved form (data not shown). However, TRAIL itself increased with AHCC (Fig 6D). Hence, Fas and TRAIL may be engaged during cell-to-cell interactions, initiating the apoptotic cascade. AML blasts have been shown to kill one another via antibody-dependent cellular cytotoxicity (ADCC) after treatment with IFNγ [29], so this AHCC-mediated Fas and TRAIL upregulation may represent a separate mechanism by which AML blasts can be induced to target one another.

### AHCC is not toxic toward healthy monocytes

**Fig 7:**
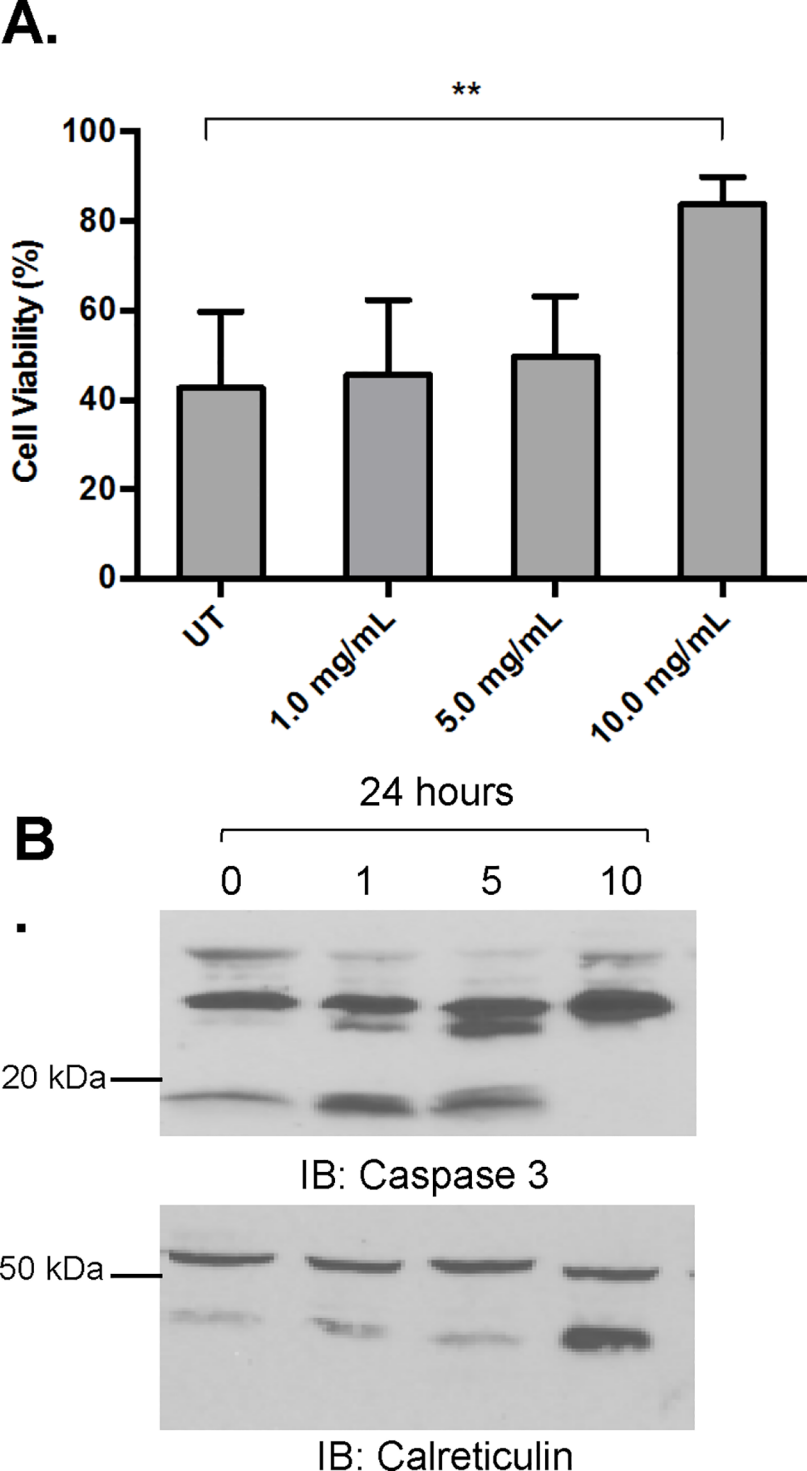
AHCC is not toxic toward healthy monocytes. Healthy-donor monocytes (5 x 10^6^ cells/ml, n = 3 donors) were treated with 0, 1, 5 or 10 mg/ml of AHCC for 24 hours. (A). Trypan Blue counts were done to measure viability. (B). Western blotting was done to measure cleaved Caspase-3, using Calreticulin as the loading control. Representative blots shown. ** p≤0.01.

Because AHCC reduced viability in AML cells, we next tested whether it had similar effects on more fully-developed myeloid-lineage cells. For this we treated primary healthy-donor monocytes with increasing doses of AHCC as above. Cell viability and Caspase-3 cleavage were both measured. In sharp contrast to its effects on AML cells, AHCC increased cell viability at 10 mg/ml (Fig 7A) and decreased Caspase-3 cleavage (Fig 7B). This selective cytotoxic effect of AHCC may suggest that it targets certain molecules and / or pathways found in AML blasts but not in mature, healthy myeloid-lineage cells.

### AHCC increases survival *in vivo*

**Fig 8:**
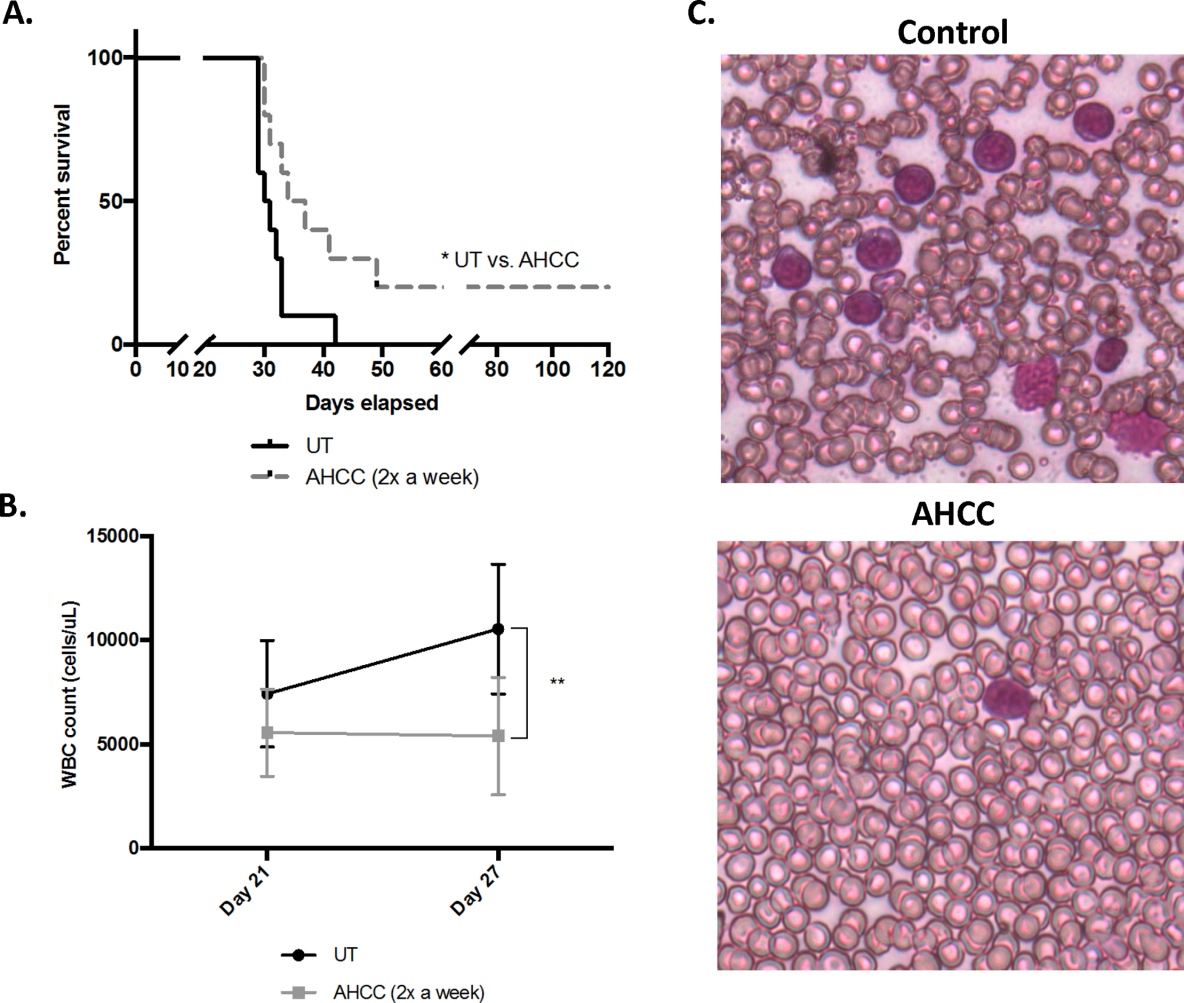
AHCC increases survival *in vivo*. NSG mice (n = 10 per group) were intravenously injected with 0.3 x 10^6^ MV4-11 cells, then monitored for one week. Mice were then treated with either PBS or AHCC (600 mg/kg) twice a week for two weeks via gavage. (A). Survival time was measured and plotted. (B). Peripheral blood was collected and white-blood-cell (WBC) counts done at day 21 and day 27 (n = 3 mice per group). (C). Wright-Giemsa stains of peripheral blood was performed at day 27 to visualize blast count and morphology, for control (top panel) and AHCC-treated (bottom panel) mice. * p≤0.05; ** p≤0.01.

AHCC induced both cleaved Caspase-3 and AML-cell death *in vitro*. Here, we tested whether AHCC could increase survival time in a murine model of AML. For this, we injected human MV4-11 cells intravenously into NSG mice, waited one week to permit engraftment, then gavaged mice twice per week for 2 weeks with either AHCC or PBS. Results showed that mice treated with AHCC survived significantly longer than those receiving PBS (Fig 8A). White-blood-cell counts were also taken on Days 21 and 27 (before and after disease symptoms appeared), and results showed that the AHCC-treated mice had significantly fewer WBC at Day 27 compared to untreated mice (Fig 8B and 8C). Hence, AHCC antagonizes AML blasts not only *in vitro*, but also *in vivo*.

## Discussion

In this study, we demonstrate that AHCC has a direct effect on AML blasts, reducing viability and proliferation in AML cell lines and in primary AML samples. AHCC drove a pro-apoptotic signal that appeared dependent at least in large part on Caspase-3 activation, as blocking Caspase-3 restored AML-cell viability. AHCC also decreased white-blood-cell counts and increased survival in a murine engraftment model of AML.

AHCC consists of a mixture of various compounds, and the mechanisms by which it acts have not been fully elucidated. It is thought that TLR4 and possibly TLR2 may play a role in AHCC induced immune responses in intestinal epithelial cells [36]. Here, at least with regard to AML cells we found that AHCC leads to activation of Caspase-3, which in turn induces apoptosis. Caspase-3 is a part of the family of executioner caspases (along with Caspases 6 and 7), which form inactive pro-caspase dimers. Once activated, these dimers are cleaved by initiator caspases into large and small subunits, allowing the two active sites of the dimer to become a mature protease [37]. Once Caspase-3 is cleaved into a mature protease, it can then initiate the apoptotic process. The activation of Caspase-3 can be initiated by both an extrinsic and intrinsic stimulus. The extrinsic apoptotic pathway is activated by the binding of a ligand to its death receptor, whereas the intrinsic pathway can be activated through various cellular stresses resulting in the release of cytochrome c [38]. In the extrinsic pathway, once the death receptor binds to its ligand, the initiator Caspase-8 becomes activated. Activated Caspase-8 can then directly cleave and activate downstream effector caspases including Caspase-3, triggering apoptosis [39]. Our results suggest that AHCC triggered this extrinsic pathway as it upregulated Fas and TRAIL, with accompanying Caspase-8, but not Caspase-9 cleavage.

Although not widely popular in the west, AHCC has been used as a nutritional supplement throughout Japan and Asia for the last decade [14] and appears well-tolerated [40]. The extract has previously been shown to have antitumor effects. For example, it prolongs survival in advanced liver cancer patients [19], enhances the antitumor activity of 5-fluorouracil [41], and reduces tumor burden alone and in combination with CpG oligodeoxynucleotides in a murine melanoma model [17]. It shows immune-modulatory effects as well such as serving to sooth hapten-induced colitis in rats [42], to decrease bacterial burden [43], and to enhance resistance to pathogens such as Chlamydia trachomatis in murine models of stress [26]. As such, AHCC may not only aid in the clearance of AML blasts, it may also help reduce the incidence of infections in these typically immune-compromised patients.

Perhaps as importantly, AHCC has been shown to reduce the adverse effects seen with chemotherapeutic agents [44–47]. Current AML therapies largely consist of intensive chemotherapy and allogeneic hematopoietic stem cell transplantation, but outcomes in elderly patients are especially poor due to their inability to receive intensive chemotherapy [48]. Hence, AHCC may enable these treatments to be extended to the elderly population. Younger patients may also benefit, especially if the AML-clearing effects of AHCC can be borne out in future clinical trials. Along with this, newer drugs such as hypomethylating agents are emerging and immune-based therapies have already shown great promise for other types of malignancies [49]. AHCC likely will not be curative for AML by itself, but still might provide powerful antitumor effects in combination with one or more of these therapies. The potential protective and / or antitumor effects of AHCC in combination with chemotherapeutic agents has been shown to have positive effects within the context of solid tumors including pancreatic, ovarian, lung, colorectal and breast cancer [45–47]. Our results suggest that it may be particularly effective for AML, as it appears to directly induce blast-cell apoptosis without harming later-lineage monocytes.

Although not surprising given the low toxicity seen with AHCC, it is nevertheless interesting that AHCC led to apoptosis in AML cells but not their healthy-donor monocyte counterparts. It is known that the metabolic needs and intracellular signaling profiles of tumor cells differ from those of normal cells [50], and one or more compounds within AHCC might exploit this to effect Caspase-3-mediated apoptosis. Alternatively, tumor cells may express more AHCC-binding molecules and thereby bind one or more of the AHCC components. Our initial tests using MV4-11 cells opened the possibility that FLT3 signaling may sensitize the cells to AHCC, as they carry the FTL3-ITD mutation [51]. However, OCI-AML3 is FTL3-WT [51] and showed similar Caspase-3 cleavage and apoptosis. Of particular interest, THP-1 cells, also FLT3-WT [52], showed no Caspase-3 cleavage and virtually no signs of apoptosis. The THP-1 cell line carries a t(9;11)(p22;q23) which leads to an MLL-AF9 fusion gene, whereas the FLT3-WT and AHCC-sensitive OCI-AML3 cells carry an NPM1-mutation [53,54]. *In vitro*, this fusion protein causes an increase in the expression of both migration and invasion genes in hematopoietic stem cells, driving an extremely invasive subtype of AML. Additionally, the transplantation of retrovirally-expressing MLL-AF9 hematopoietic cells into mice causes rapidly-progressing disease when compared to control mice [55]. The other two cell lines that responded to AHCC do not carry this specific translocation [56,57], suggesting that it could be a factor with regard to AHCC resistance. However, numerous mutations and mutational combinations exist within AML cells and cell lines that might influence their response to AHCC. Differential screens between resistant versus sensitive cell lines may help uncover the molecules and signaling pathways that are targeted by AHCC, and perhaps which AML subtypes may be most responsive.

The mutational status of 5 of the 7 patient samples we tested represented 3 different mutational classes, including FLT3-ITD, FLT3-TKD and NPM1 (S1 Table). All responded to AHCC, suggesting that its effect is likely independent of FLT3 and NPM1. Due to the large number of known mutations and cytogenetic profiles, as well as patient-to-patient variability, very large sets of patient samples will be required to determine which are associated with response to AHCC.

In summary, we have found that AHCC can cause Caspase-3-dependent AML cell death in both MV4-11 cells and primary AML samples. It also increased survival time in a murine engraftment model. Hence, the study of AHCC as a potential adjuvant for the treatment of AML may be warranted.

## Supporting information

S1 Table Mutational status of AML patients. De-identified AML patient samples provided through the Leukemia Tissue Bank (LTB) at The Ohio State University were tested for FLT3-ITD, FLT3-TKD, NPM1, CEBPα, BCR-ABL and PML-RARα. The symbols “-”indicate negative and “+” positive for each respective mutation. 2 of the 7 patient sets had not been tested by the LTB.
(PDF)
