# Supplementary material for: Active hexose-correlated compound enhances extrinsic-pathway-mediated apoptosis of Acute Myeloid Leukemic cells
Source: PLoS One. 2017 Jul 20;12(7):e0181729. doi: 10.1371/journal.pone.0181729 (PMC5519206; doi:10.1371/journal.pone.0181729)
Supplement: S1 Table — De-identified AML patient samples provided through the Leukemia Tissue Bank (LTB) at The Ohio State University were tested for FLT3-ITD, FLT3-TKD, NPM1, CEBPα, BCR-ABL and PML-RARα. The symbols “-”indicate negative and “+” positive for each respective mutation. 2 of the 7 patient sets had not been tested by the LTB. (PDF) [file pone.0181729.s001.pdf]

Sample ID	FLT3 ITD	FLT3 TKD	NPM1	CEBPα	BCR-ABL	PML-RARα
U-13-1982	-	+	+	-	-	-
U-14-0465	(not tested)	(not tested)	(not tested)	(not tested)	(not tested)	(not tested)
U-14-0269	(not tested)	(not tested)	(not tested)	(not tested)	(not tested)	(not tested)
U-13-1355	+	-	+	-	-	-
U-13-1072	+	-	+	-	-	-
U-14-1295	-	+	-	-	-	-
U-14-2181	+	-	+	-	-	-

S1 Table. Mutational status of AML patients.
